# Propylene Polymerization Catalyzed by Metallocene/Methylaluminoxane Systems on Rice Husk Ash

**DOI:** 10.3390/molecules24081467

**Published:** 2019-04-13

**Authors:** Kuo-Tseng Li, Cheng-Ni Yang

**Affiliations:** Department of Chemical Engineering, Tunghai University, Taichung 40704, Taiwan; vinini@taiwancement.com

**Keywords:** propylene polymerization, rice husk ash, supported metallocene catalysts, polypropylene, turbulent mixing

## Abstract

Silica generated from agricultural waste is more cost effective and environmentally friendly than silica from traditional commercial processes. In this study, spherical silica particles with a diameter of around 120 nm were fabricated from rice husk ash (RHA), and were used to support two bridged zirconcene complexes ((I) Me_2_Si(Ind)_2_ZrCl_2_ and (II) C_2_H_4_(Ind)_2_ZrCl_2_) for catalyzing propylene polymerization to produce polypropylene (PP) in a temperature range of 40–70 °C and in a solution methylaluminoxane (MAO) range of 0.1–0.6 wt%. Due to its small particle size, RHA-supported catalyst exhibited much higher activity than micro-sized commercial silica-supported catalyst. At the optimum polymerization temperature of 55 °C and with increasing MAO concentration, polymer yield increased proportionally with the increase of number average molecular weight. Compared to (I), (II) produced more polymer molecules but with much shorter chain length, ascribed to the differences of Zr loading and bridge structure. With increasing polymerization temperature, polymer molecular weight decreased rapidly and resulted in a significant change of PP assembly morphology (shape and size). At 55 °C, (I) produced uniform PP assemblies which had dumbbell-like structure with a smooth middle section and two fibrillar ends, while (II) produced spherical PP particles. The dumbbell middle part width was essentially identical to the Batchelor microscale proposed in turbulent mixing theory.

## 1. Introduction

Polypropylene (PP) is a flexible product which can be used in many industries, including packing, automobile, toy making, carpeting, plastic paper, and laboratory equipment manufacturing [1,2,3,4]. It accounts for one quarter of total plastics made today [5] with a global demand of around 60 million tons in 2015 and is the second largest polymer business in the world [6]. Traditionally, MgCl_2_-supported Ziegler–Natta catalysts are used for producing PP in a continuous slurry reactor or in a gas fluidized bed reactor. However, metallocene/methylaluminoxane (abbreviated as MAO) catalyst systems exhibit much higher activity and produce PP with higher purity and more types of microstructures than Ziegler–Natta catalysts [1,7].

In order to produce morphologically uniform polymer particles with high bulk density and to avoid reactor fouling, industrial metallocene catalysts have to be supported on a MAO-modified solid carrier (so-called drop-in catalysts) [8]. Micro-sized amorphous and porous silica is most commonly used for supporting metallocence/MAO catalysts because of its high surface area/porosity, good mechanical properties, and fine stability under reaction and processing conditions [9,10].

Commercial porous silica is made by a multi-step process with aqueous alkali metal silica as the precursor [8], which makes it less cost effective and not very environmentally friendly. Thus, there is a need to find a less expensive and more environmentally friendly silica precursor. Naturally occurring silica, especially those found in agro waste, can provide an alternative source to replace commercial silica precursors [11]. Rice husk is an agricultural waste material abundantly available in rice-producing countries, containing 20% ash, 38% cellulose, 22% lignin, 18% pentose, and 2% other organic compounds [12,13]. Its ash has more than 94% silica, SiO_2_, content [13]. Rice husk-derived silica has been used recently to support a zirconcene/MAO for catalyzing ethylene polymerization [14].

In this study, silica with a particle size of around 120 nm was fabricated from rice husk and was used to support two metallocenes (Me_2_Si(Ind)_2_ZrCl_2_ and C_2_H_4_(Ind)_2_ZrCl_2_) for catalyzing propylene polymerization, which exhibited much higher polymerization activity than commercial micro-sized silica-supported catalyst. The effects of liquid MAO concentration, metallocene, reaction temperature, and agitation speed were investigated, and these variables showed strong influence on polymer yield, properties, and the morphology of polymer assembly produced.

## 2. Results and Discussion

### 2.1. Rice Husk Ash (RHA) and Catalyst

**Figure 1:**
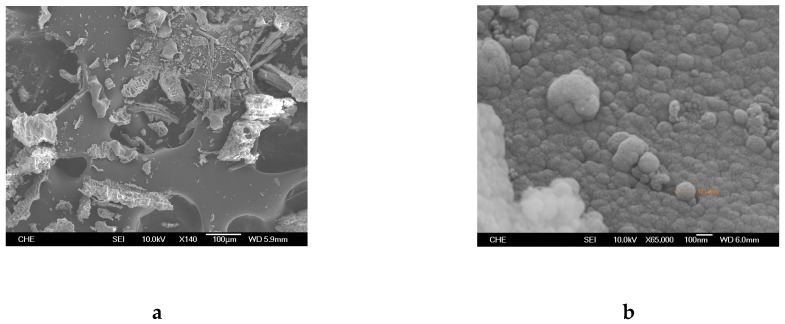
Scanning electron microscopy (SEM) images of rice husk ash with magnifications of (**a**) 140 and (**b**) 65,000.

Figure 1 presents scanning electron microscopy (SEM) images of RHA with the magnifications of 140 and 65,000. Figure 1a shows that RHA maintains the rice husk shape after calcination at 700 °C, and contains a huge number of sphere silica particles with a diameter of around 120 nm, as shown in Figure 1b.

**Scheme 1:**
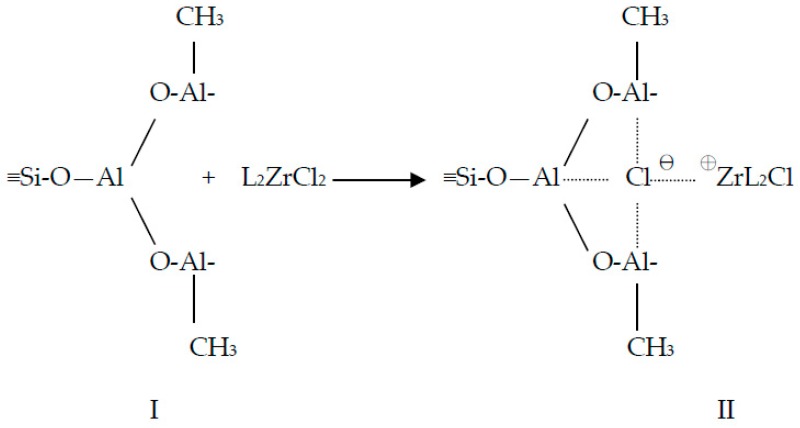
Generation of species II form MAO modified silica (species I) and metallocene.

Inductively coupled plasma-atomic emission spectrometer (ICP-AES) measurements indicated that MAO-treated RHA had an Al content of around 1.2 wt%, the RHA-supported Me_2_Si(Ind)_2_ZrCl_2_ and C_2_H_4_(Ind)_2_ZrCl_2_ catalysts had Zr contents of 0.39 wt% and 0.68 wt% (i.e., [Al]/[Zr] atomic ratios were 10.3 and 5.9), respectively, suggesting that C_2_H_4_(Ind)_2_ZrCl_2_ had greater adsorption ability than Me_2_Si(Ind)_2_ZrCl_2_ (because the metallocene amount used was identical and these two metallocene have similar molecular weight, 418.47 g/mol vs. 448.57 g/mol). In Scheme 1, species II is generated from the adsorption of L_2_ZrCl_2_ complex (L = Me_2_Si(Ind)_2_ or C_2_H_4_(Ind)_2_) on the MAO-modified silica (species I) [15,16]:

where species I is produced from the immobilization of MAO on silanol (Si-OH).

These Al and Zr contents of RHA were much lower than those obtained before with the use of a commercial micrometer-sized silica [16,17], which had an Al content of around 5 wt%, Zr contents of ~1 wt% and 1.5 wt% for the commercial silica-supported C_2_H_4_(Ind)_2_ZrCl_2_ and Me_2_Si(Ind)_2_ZrCl_2_ catalysts, respectively. That is, Al content of MAO-treated RHA is around one quarter of that obtained with MAO-treated commercial silica, which should be mainly due to their difference in silanol groups (≡SiOH), caused by the difference of heat treatment temperature (700 °C for RHA and 450 °C for commercial silica). It has been reported that hydroxyl group concentration on amorphous silica surface were 1.1 and 2.0 OH groups/nm^2^ after vacuum treatment at 700 °C and 450 °C, respectively [18]. The high heat-treatment temperature (700 °C) is necessary to remove all organic compounds and carbon residual from RHA surface.

### 2.2. Propylene Polymerization and Polymer Characterization

#### 2.2.1. Effect of Solution MAO Concentration

**Figure 2:**
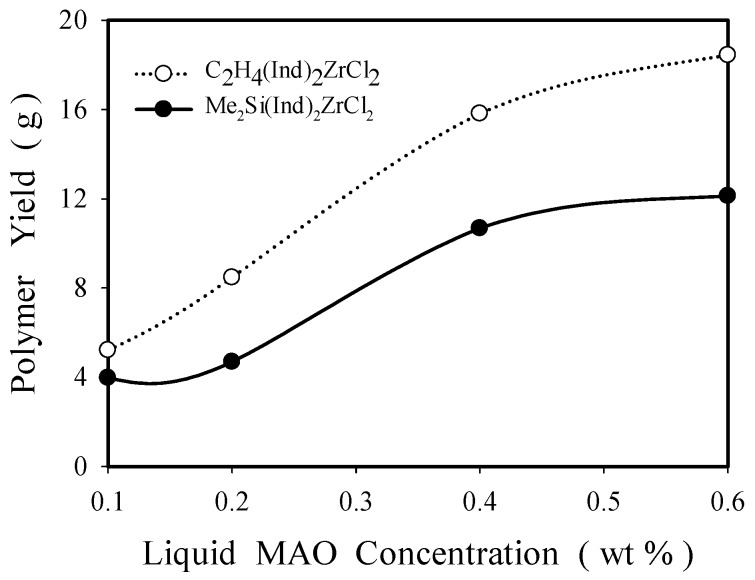
Effects of methylaluminoxane (MAO) concentration on polymer yield.

Solution MAO concentration effect was investigated at 55 °C polymerization temperature and 400 rpm agitation speed. With the increase of solution MAO concentration from 0.1 wt% to 0.6 wt%, Figure 2 shows that polymer yield increases continuously from 4.0 g to 12 g and from 5.2 g to 18.4 g for RHA-supported Me_2_Si(Ind)_2_ZrCl_2_ and C_2_H_4_(Ind)_2_ZrCl_2_, respectively. That is, the latter exhibits significantly higher polymer yield than the former, which should be partly due to the higher Zr loading of the latter (0.68 wt% for the latter and 0.39 wt% for the former, as mentioned above). When solution MAO concentrations were 0.4 wt% and 0.6 wt%, the respective polymerization activities obtained with Me_2_Si(Ind)_2_ZrCl_2_ were 4142.7 and 4701.3 kg PP/mol Zr.h, which were 1.17 and 1.14 times those obtained with C_2_H_4_(Ind)_2_ZrCl_2_. That is, polymerization activity based on the number of Zr atom was not sensitive to the change of metallocene type. For copolymerization of ethylene with 1-hexene, Ouijada et al. also found that three different types of metallocenes had similar catalytic activities [19].

**Figure 3:**
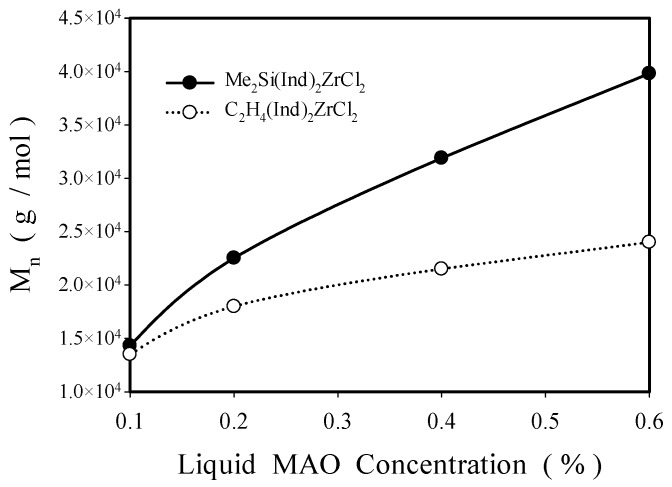
Influence of metallocene and MAO concentration on number average molecular weight (M_n_).

With the increase of MAO concentration, number average molecular weight (M_n_) of polymers increases continuously for both metallocenes, as shown in Figure 3. In addition, polymers produced with Me_2_Si(Ind)_2_ZrCl_2_ have much greater molecular weight than those produced with C_2_H_4_(Ind)_2_ZrCl_2_, especially in the range of high MAO concentration. At MAO concentrations of 0.1, 0.2, 0.4, and 0.6 wt%, polymers produced with Me_2_Si(Ind)_2_ZrCl_2_ had polydispersity indexes (= M_w_/M_n_) of 3.4, 2.3, 2.5, and 2.7, respectively; polymers produced with C_2_H_4_(Ind)_2_ZrCl_2_ had polydispersity indexes of 3.2, 2.1, 2.0, and 2.2, respectively. PP steroregularity is represented by *mmmm* pentad content ([*mmmm*]). ^13^C-NMR measurements indicated that the percent *mmmm* pentads were around 90% and 85% for polymers produced at 55 °C with Me_2_Si(Ind)_2_ZrCl_2_ and C_2_H_4_(Ind)_2_ZrCl_2_ catalysts, respectively, and are insensitive to the change of liquid MAO concentration.

**Scheme 2:**
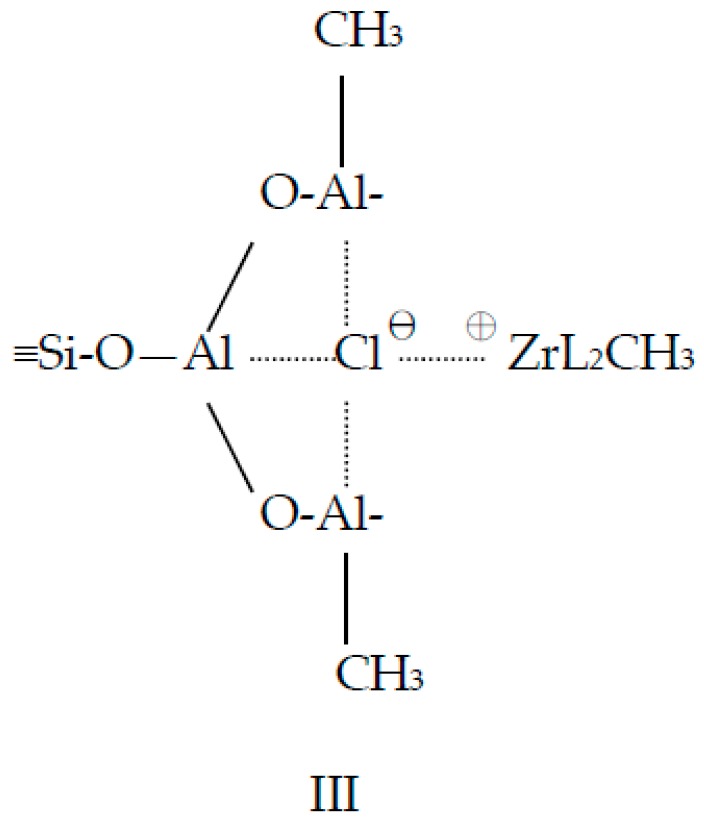
Chemical structure of species III.

Bonine et al. [20] found that chemical kinetic scheme for propylene polymerization on silica-supported metallocene catalyst was in agreement with unsupported (i.e., homogeneous) catalyst, which includes active site generation, propagation, β–H transfer, reactivation, and deactivation. Therefore, for the polymerization of propylene (with a molecular weight of 42 g/mol), M_n_ can be calculated from the following equation [21,22]:

M_n_ = 42/[(k_TM_/k_P_) (1/[C]) + k_TO_/k_p_] (1)

where [C] is the concentration of propylene, k_P_ is the rate constant of propagation steps, k_TM_ is the rate constant of chain termination step due to β–H transfer to the metal, and k_TO_ is the rate constant of chain termination step due to β–H transfer to an olefin. C_2_H_4_(Ind)_2_ZrCl_2_ might have greater values of k_TM_ or k_TO_ than Me_2_Si(Ind)_2_ZrCl_2_, because the ethyl bridge is more flexible, longer, and more open than the dimethylsiyl bridge, which resulted in its lower polymer molecular weight and isotacticity. The combination of Figure 3 and Equation (1) also indicates that rate constant ratios k_TM_/k_P_ or k_TO_/k_P_ increase significantly with the increase of MAO concentration. In the presence of liquid MAO, ZrL_2_CH_3_ (species III) was formed from the methylation of species II (in Scheme 1) with liquid MAO [15,16], which increases species III (with a structure shown in Scheme 2) concentration and decreases species II concentration. The results in Figure 3 (PP molecular weight increases with increasing MAO concentration) suggest that species III has higher ratios of k_TM_/k_P_ or k_TO_/k_P_ than species II.

The results in Figure 3 are in agreement with those observed by Ghiotto et al. [23], who studied the influence of MAO concentration on the performances of 1-hexene polymerization using homogeneous *rac*-Me_2_Si[2-Me-BenzeneInd] _2_ZrCl_2_ as the catalyst precursor. They found that toluene solution contained 30 wt% Al compounds (denoted as MAO-30), and produced polymers with higher yield and greater molecular weight (71,000 g/mol vs. 51,000 g/mol) than toluene solution that contained 10 wt% Al compounds (denoted as MAO-10). They also found that the former had a higher apparent propagation rate constant (k_p_^app^, 0.34 L/mol.s vs. 0.17 L/mol.s) than the latter. With the use of a much more complicated kinetic model, they obtained that the former had a lower intrinsic propagation rate constant (k_p_, 1.05 L/mol.s vs. 1.28 L/mol.s) and lower termination rate constant (k_t_, 3.42/s vs. 1.91/s). Therefore, the effect of MAO concentration on PP molecular weight (shown in Figure 3) might be primarily due to the decrease of termination rate constants with increasing MAO concentration.

However, the results in Figure 3 are opposite to those observed by Hung and Rempel [24], who studied propylene polymerization using homogeneous Et[H_4_Ind]_2_ZrCl_2_/MAO catalysts and found that M_n_ decreased with increasing [Al]/[Zr] ratio. They ascribed their results to the chain transfer to MAO and the frequency of this chain transfer was proportional to MAO concentration.

**Figure 4:**
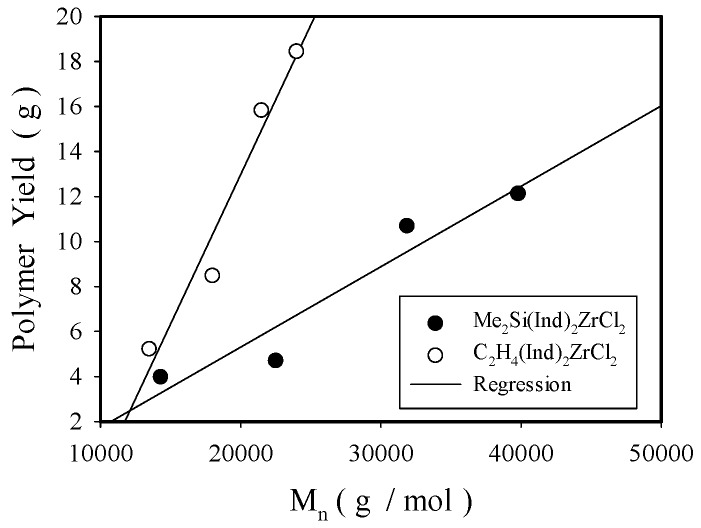
Relationships between polymer yield and number average molecular weight.

Figure 4 shows that there are approximately linear relationships between polymer yield and number average molecular weight for all polymers produced with C_2_H_4_(Ind)_2_ZrCl_2_ and Me_2_Si(Ind)_2_ZrCl_2_, indicating that the increases of polymer yield with increasing MAO concentration are due to the increase of polymer molecular weight, not due to the increase of the number of polymer molecule produced or the change of the active site number for propylene polymerization.

In Figure 4, the regression line slopes represent the moles of a polymer produced with 0.03 g catalyst, which are 3.57 × 10^−4^ and 1.4 × 10^−3^ for Me_2_Si(Ind)_2_ZrCl_2_ and C_2_H_4_(Ind)_2_ZrCl_2_, respectively. That is, the latter produced four times as much polymer molecules as the former did, which should be due to the difference of Zr loading (Zr content of the latter was 1.8 times that of the former) and bridge structure (C_2_H_4_ vs. Me_2_Si).

**Figure 5:**
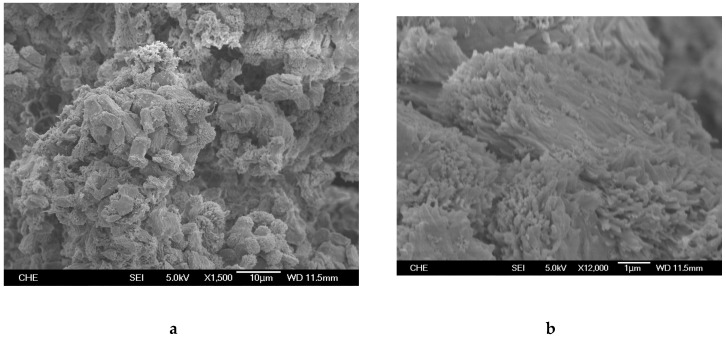
Field emission (FE)-SEM pictures of polypropylene (PP) particles produced with Me_2_Si(Ind)_2_ZrCl_2_ at 0.4 wt% solution MAO concentrations and 55 °C with the magnifications of (**a**) 1500 and (**b**) 12,000.

Figure 5 presents SEM pictures of PP assemblies produced with RHA-supported Me_2_Si(Ind)_2_ZrCl_2_ at 0.4 wt% MAO concentration and 55 °C with magnifications of 1500 and 12,000. It can be clearly seen from Figure 5a that most PP assemblies exhibit the shape of a dumbbell with a smooth middle section and two fibrillar ends. The close-up view of PP particles in Figure 5b clearly indicates that each PP assembly is packed with numerous nano-sized PP fibrils. Schaer et al. [25] studied silica particle aggregation in a batch-agitated vessel and found that a perikinetic aggregation mechanism occurred for silica particles smaller than 250 nm where silica collisions are brought about by Brownian motion (i.e., diffusion) of very small-scale particles. Therefore, it is possible that RHA with a diameter of about 120 nm aggregated in a fluid element where molecular diffusion is important (i.e., Batchelor microscale). It is known that propylene polymerization is highly exothermic with a reaction heat of about 16.5 kcal/mol [26]; the smooth middle section in Figure 5a suggests that catalytic active sites for polymerization are located in this section, which had the highest local temperature and therefore greatest PP solubility in toluene due to the large propylene polymerization heat released at the active sites. Figure 5 also indicates that PP molecules grew from the active sites in the middle section toward two ends of the assembly, which resulted in the numerous PP fibrils observed at two ends of the dumbbell-like assemblies.

The width of PP assembly middle section in Figure 5 is around 4 μm, which is essentially identical to the calculated Batchelor microscale proposed in turbulent theory (~3.6 μm under the reaction condition [22]). There are two important length scales proposed in turbulent mixing theory: The Kolmogorov scale (η_K_= (ν^3^/ε)^1/4^) and the Batchelor microscale (η_B_ = (ν D_AB_^2^/ε)^1/4^), where ν is kinematic viscosity of solution, D_AB_ is diffusion coefficient of A in B, and ε is the turbulent kinetic energy dissipation rate of per unit mass [27,28]. The Kolmogorov scale is the turbulence scale where viscosity becomes dominant and the Batchelor microscale is the length scale where molecular diffusion becomes important [29,30,31]. The similarity between PP assembly middle-section width and the Batchelor microscale suggests that the dumbbell-shape polymer assemblies in Figure 5 should be fabricated inside fluid elements with a size of Batchelor microscale, where propylene diffusion in toluene becomes important.

**Figure 6:**
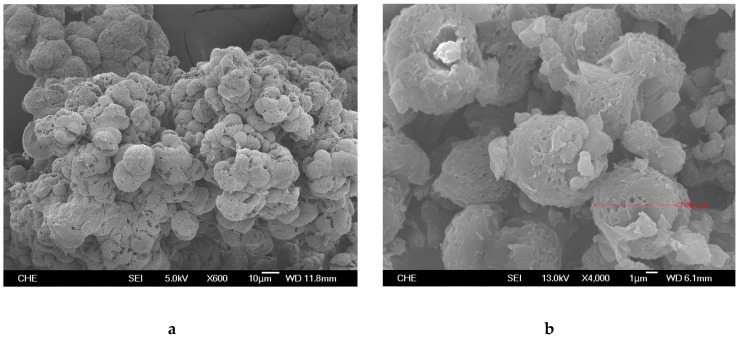
FE-SEM pictures of PP particles produced with C_2_H_4_(Ind)_2_ZrCl_2_ at 0.4 wt% solution MAO concentrations and 55 °C at agitation speeds of (**a**) 400 rpm and (**b**) 100 rpm.

Figure 6 presents SEM images of PP particles produced with RHA-supported C_2_H_4_(Ind)_2_ZrCl_2_ at 0.4 wt% MAO concentration and 55 °C using two different agitation speeds: 400 rpm and 100 rpm. It is interesting to note that PP particle shape and size in Figure 6a are completely different from those in Figure 5a, which might be caused by the differences of polymer yield (shown in Figure 2), polymer molecular weight (shown in Figure 3), and isotacticity produced with these two different metallocenes. As shown in Figure 2 and Figure 3, C_2_H_4_(Ind)_2_ZrCl_2_ had higher polymer yield and produced PP with lower molecular weight and isotacticity than Me_2_Si(Ind)_2_ZrCl_2_, which resulted in the greater solution viscosity (because PP solubility in toluene increases with decreasing polymer molecular weight and isotacticity) and therefore the lower Reynolds number (Re is inversely proportional to kinematic viscosity) for the C_2_H_4_(Ind)_2_ZrCl_2_ reaction mixture. The decrease of the Reynolds number resulted in the dramatic change of polymer assembly shape and size because the flow conditions changed from a turbulent region for producing PP in Figure 5 to a non-turbulent region for producing PP in Figure 6a. PP assembly shape in Figure 6a is similar to the rice husk ash shape in Figure 1b, suggesting that RHA acted as the template for forming PP assembly under the condition.

With the decrease of agitation speed from 400 rpm to 100 rpm, polymer yield changed from 15.8 g to 11.2 g, suggesting that polymerization rate was strongly affected by external mass transfer of propylene to catalyst surface. It is known that reaction rate is directly proportional to the square root of velocity (=agitation speed × impeller diameter) under external mass transfer limited conditions [32]. PP particles formed at the agitation speed of 100 rpm (in the non-turbulent region) are also spherical in shape with a diameter of around 7 μm, as shown in Figure 6b.

**Figure 7:**
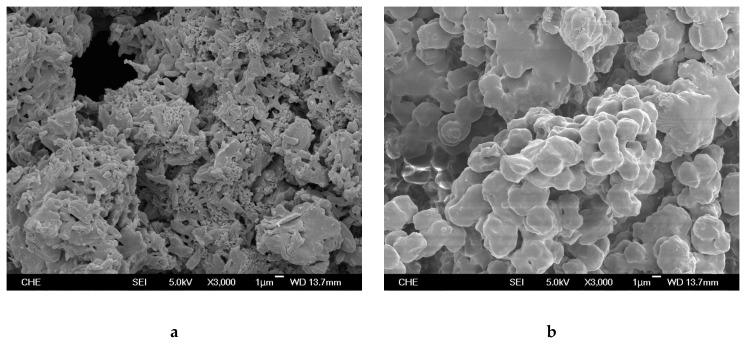
FE-SEM pictures of PP particles produced with homogeneous (**a**) Me_2_Si(Ind)_2_ZrCl_2_ and (**b**) C_2_H_4_(Ind)_2_ZrCl_2_ catalysts at 0.2 wt% solution MAO concentrations and 55 °C at an agitation speed of 400 rpm.

Figure 7 shows SEM pictures of PP particles produced with 0.0007 g homogeneous Me_2_Si(Ind)_2_ZrCl_2_ and C_2_H_4_(Ind)_2_ZrCl_2_ at 0.2 wt% MAO concentration, 400 rpm, and 55 °C. Polymer obtained with homogeneous Me_2_Si(Ind)_2_ZrCl_2_ was 11.5 g. Comparison of Figure 5a and Figure 7a indicates that supported Me_2_Si(Ind)_2_ZrCl_2_ catalysts produced PP particles with much more uniform shape and size than homogeneous catalyst. Polymer obtained with homogeneous C_2_H_4_(Ind)_2_ZrCl_2_ was 6.9 g. Comparison of Figure 6b and Figure 7b indicates that supported C_2_H_4_(Ind)_2_ZrCl_2_ can produced discrete PP particles with greater size while PP particles produced with homogeneous C_2_H_4_(Ind)_2_ZrCl_2_ glue together.

#### 2.2.2. Effect of Polymerization Temperature

**Figure 8:**
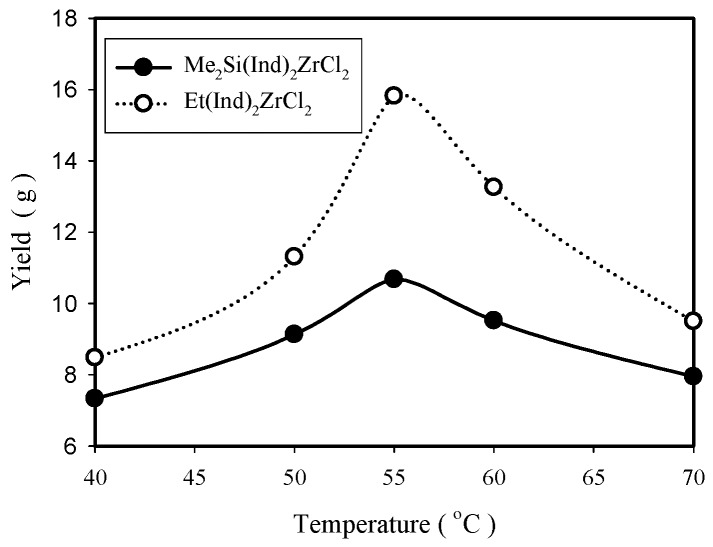
Influence of reaction temperature and metallocene on polymer yield.

Polymerization temperature effect was studied at 0.4 wt% MAO concentration and 400 rpm agitation speed. Figure 8 shows that polymer yield is very sensitive to the change of polymerization temperature for both RHA-supported Me_2_Si(Ind)_2_ZrCl_2_ and C_2_H_4_(Ind)_2_ZrCl_2_ catalysts, which have the same optimum reaction temperature at 55 °C with the respective maximum polymer yields of 10.7 g and 15.8 g.

For propylene polymerization on silica-supported metallocene/MAO catalysts, each active site involves propagation reaction of first order with respect to both monomer and site [20]. That is, the rate of polymerization R_p_ is given as [23]:

R_p_= k_p_^app^ [C^*^] [M] (2)

where [C^*^] and [M] are concentration of catalytically active species and concentration of propylene in toluene, respectively. k_p_^app^ is the apparent propagation rate constant. It is known that gas solubility in liquid decreases with increasing temperature and propylene solubility in toluene ([M] in Equation (2)) decreased linearly with increasing temperature [33]. Based on material balance, [C^*^] in Equation (2) is related to total zirconcene concentration [Zr] according to the following equation [23]:

[C^*^] + 2[C2] + [C-MAO] + [C_d_] = [Zr] (3)

where C2 and C-MAO are two types of inactive species, which are in dynamic equilibrium with C^*^, C_d_ is the dead species resulting from irreversible catalyst deactivation and

[C_d_] = ∫(k_d_ [C^*^]/[MAO])dt. (4)

With the increase of polymerization temperature, k_p_ and k_d_ increased but both [M] and [C^*^] decreased (due to the increase of C_d_). When the polymerization temperature was ≤50 °C, k_p_^app^ increased more rapidly than the decrease of [C^*^] and [M], which resulted in the increase of the observed polymerization activity. With increasing reaction temperature, k_p_^app^ increases but both [C^*^] and [M] decrease [16,17]. Figure 8 indicates that the decrease of [C^*^] [M] is more significant than the increase of k_p_^app^ when polymerization temperature is greater than 55 °C.

**Figure 9:**
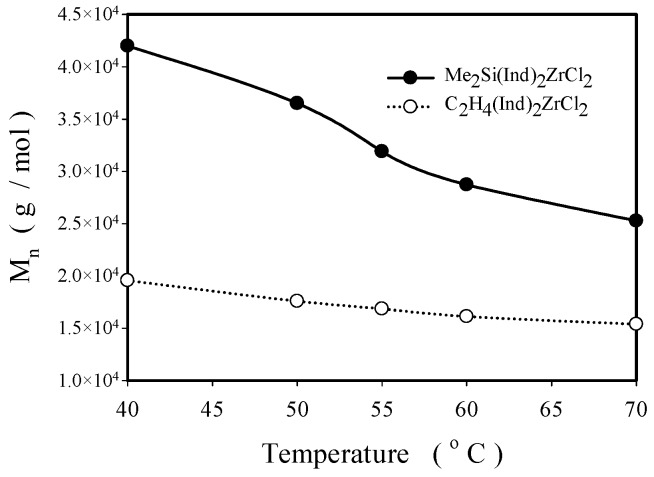
PP number average molecular weight vs. reaction temperature.

Figure 9 shows that polymer molecular weight decreases rapidly with increasing polymerization temperature, which should be due to the enhanced chain-transfer reaction rate because k_TM_ and k_TO_ in Equation (1) have greater activation energies than k_P_. In the temperature range of 40–70 °C, polymer polydispersity indexes (= M_w_/M_n_) were in the range of 2.0 to 2.7, which indicates that active sites on the heterogeneous catalysts are not uniform.

**Figure 10:**
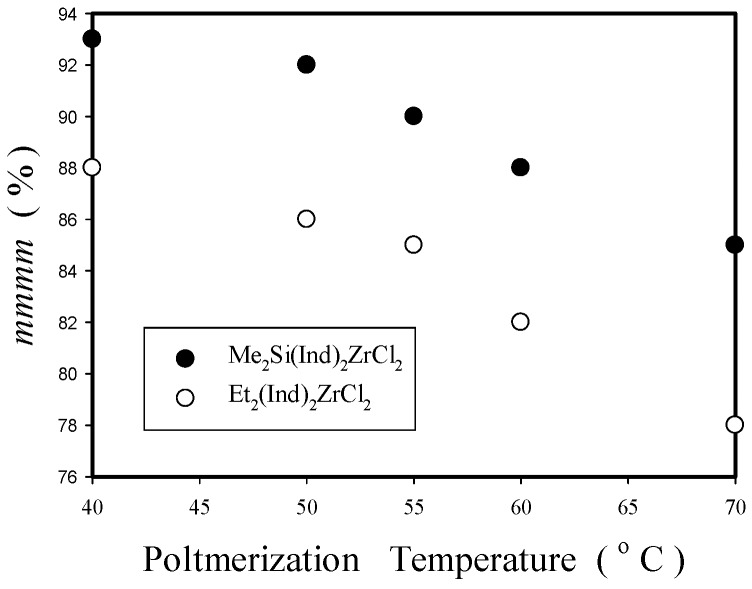
Effects of metallocene and polymerization temperature on [*mmmm*] of polymers produced.

Figure 10 shows that [*mmmm*] of the PPs made with Me_2_Si(Ind)_2_ZrCl_2_ catalyst is higher than that with the C_2_H_4_(Ind)_2_ZrCl_2_ catalyst. In addition, both PP stereoregularities decreases with increasing polymerization temperature, which might be caused by the intensified thermal disturbance of zirconocene at the higher temperature [33]. The effects of polymerization conditions on PP properties using RHA-supported Me_2_Si(Ind)_2_ZrCl_2_ catalysts are summarized in Table 1.

**Figure 11:**
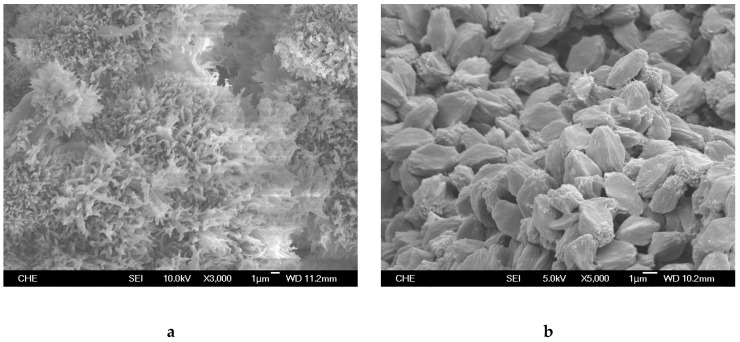
FE-SEM picture of PP particles produced with RHA-supported Me_2_Si(Ind)_2_ZrCl_2_ at (**a**) 40 °C, (**b**) 70 °C.

**Figure 12:**
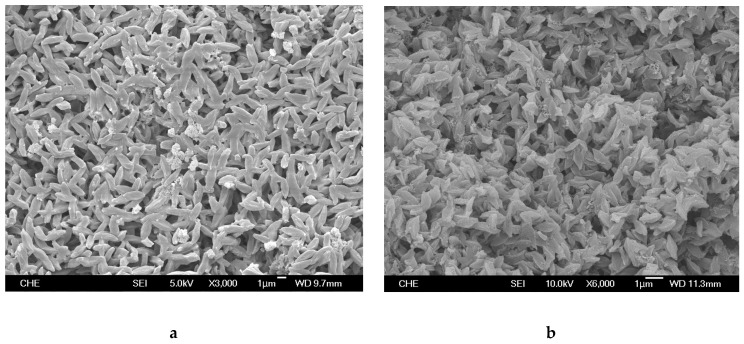
FE-SEM picture of PP particles produced with RHA-supported C_2_H_4_(Ind)_2_ZrCl_2_ at (**a**) 40 °C, (**b**) 70 °C.

Figure 11 and Figure 12 presents SEM pictures of PP assembly produced with RHA-supported Me_2_Si(Ind)_2_ZrCl_2_ and C_2_H_4_(Ind)_2_ZrCl_2_ catalysts, respectively, at the reaction temperatures of 40 °C and 70 °C. The comparisons of Figure 5 and Figure 11 (using RHA-supported Me_2_Si(Ind)_2_ZrCl_2_ as the catalyst) and Figure 6 and Figure 12 (using RHA-supported C_2_H_4_(Ind)_2_ZrCl_2_ as the catalyst) indicate that polymerization temperature has very strong influence of the polymer assembly shape and size. With the use of RHA-supported Me_2_Si(Ind)_2_ZrCl_2_, PP assembly shape changed from a discrete needle (with a diameter of around 300 nm) structure at 40 °C (Figure 11a), to a dumbbell-like structure with smooth middle section (width ~4 μm) and two fibrous ends at 55 °C (Figure 5), and finally to a pineapple shape with a middle section size of around 2 μm and smooth exterior surfaces at 70 °C (Figure 11b). The discrete PP needle structure observed in Figure 11a should be due to its high molecular weight (reported in Figure 9) which resulted in its low solubility in toluene.

Figure 12 shows that PP particles produced with RHA-supported C_2_H_4_(Ind)_2_ZrCl_2_ at 40 °C and 70 °C have similar slim rod-like shape but with significant difference in assembly size. The middle section widths are 1 μm at 40 °C (Figure 12a) and 0.5 μm at 70 °C (Figure 12b), which should be caused by the decreasing molecular weight (shown in Figure 9) and therefore shorter chain length of polymer produced with increasing reaction temperature. At the polymerization of 70 °C, polymer assembly size produced with RHA-supported Me_2_Si(Ind)_2_ZrCl_2_ (~2 μm, shown in Figure 11b) is much greater than that produced with RHA-supported C_2_H_4_(Ind)_2_ZrCl_2_ (~0.5 μm, shown in Figure 12b), which is also due to the much greater molecular weight and therefore much longer chain length of the former, as shown in Figure 9. However, PP particle shape and size in Figure 12 are very much different from those in Figure 6 (produced at 55 °C), which have a spherical shape and a diameter of around 7 μm. The difference should be primarily due to the difference of polymer yield, as shown in Figure 8.

Table 2 shows the effect of support on the performance of Me_2_Si(Ind)_2_ZrCl_2_ catalyst supported on (1) RHA (this work) and (2) a commercial micro-sized silica (diameter~100 μm, obtained from Strem Chemicals, Newburyport, MA, USA) [17]. Polymerization activity of RHA-supported catalyst is 9.4 times that produced with commercial silica-supported catalyst, which should be primarily due to the difference of silica particle size (120 nm vs. 100 μm). In addition, catalyst (1) produced polymers with greater molecular weight than catalyst (2) did.

## 3. Materials and Methods

### 3.1. Catalyst Preparation

Rice husk from Miaoli County in Taiwan was used as the precursor for producing silica particles, via the sequence of individual steps of washing (to remove dirt), acid treating (to leach out metallic components), pyrolysis (to carbonize organic components), and calcination (to burn out residual carbon) [34,35]. Pyrolysis and calcinations were carried out in a quartz tube. Details of each step are given below: (1) Wash 10 g rice husk and then dry, (2) treat the cleaned rice husk (8.8 g) with 3 N hydrochloride acid (100 mL) at 100 °C for 1 h, followed by washing (with reverse osmosis water, 9 times) and drying (at 110 °C) to obtain 5.4 g sample, (3) pyrolyze the acid-treated rice husk (3 g) at 700 °C in a quartz reactor under a nitrogen flow of 100 mL/min, (4) calcine the pyrolyzed rice husk ash at 700 °C under an air flow of 100 mL/min for 1 h to obtain 0.88 g sample.

Two metallocene/MAO catalyst systems were supported on the rice husk ash (abbreviated as RHA) obtained above, according to the following procedure [16,17]: (1) Heat 0.5 g RHA in 3.5 mL 10 wt% MAO solution at 50 °C for 24 h, then wash with 5 mL toluene three times, (2) mix the MAO-treated RHA with 0.036 g rac-diemthylsilybis(inenyl) zirconium dichloride (Me_2_Si(Ind)_2_ZrCl_2_) or rac-ethylenebis(indenyl)zirconium dichloride (C_2_H_4_(Ind)_2_ZrCl_2_) at 50 °C for 16 h, then wash with 5 ml toluene three times, and (3) dry the catalyst at 50 °C. A glove-box filled with dry argon gas was used to carry out these steps above. The 10 wt% MAO solution (in toluene) was supplied by Albemarte (Baton Rouge, LA, USA) and C_2_H_4_(Ind)_2_ZrCl_2_ and Me_2_Si(Ind)_2_ZrCl_2_ were purchased from Strem Chemicals (Newburyport, MA, USA).

### 3.2. Propylene Polymerization

The polymerization of propylene occurred in an agitated high-pressure Parr reactor with 100 mL capacity, equipped with a turbine type impeller and a temperature control system. In a typical run, 0.03 g of the above prepared RHA-supported metallocene/MAO catalysts, 0.5 to 3 mL 10 wt% MAO solution, and 50 mL toluene were charged to the reactor, which was heated to the desired temperature and then the reaction mixture was stirred at 400 rpm with a continuous supply of propylene at 100 psig. After 2 h, 1 mL acidic methanol was added to stop polymerization. The catalyst was not separated from polymer because of very low Zr concentration in the products. A vacuum oven was used to dry polymer product obtained, which was then weighted to obtain polymer yield.

### 3.3. Catalyst and Polymer Characterization

A Kontron inductively coupled plasma-atomic emission spectrometer (ICP-AES, Model S-35, Kronton, Augsburg, Germany) was used to determine catalyst composition after HF acid digestion of the solid. A JEOL scanning electron microscope (SEM, model JSM-6700) (Jeol, Tokyo, Japan) was used to observe morphology and particle size of catalyst and polymer. A Waters Alliance GPCV-2000 high-temperature gel permeation chromatograph (GPC) (Waters, Milford, MA, USA) equipped with three columns (2 Styragel HT6E and 1 Styragel HT2) and with a mobile phase of *O*-dichlorobenzene at 135 °C was used to measure polymer molecular weight and molecular weight distribution. Polystyrene standards were used to establish the calibration curve. Polymer tacticity was determined from Carbon 13 NMR spectra, measured at 100 °C in 1,2-dichlorobenzene-d4 solvent with a Varian UNITY-600 NMR spectrometer (Varian, Palo Alto, CA, USA).

## 4. Conclusions

MAO-treated rice husk ash (RHA) contained 120 nm spherical silica particles, was used to support Me_2_Si(Ind)_2_ZrCl_2_ and C_2_H_4_(Ind)_2_ZrCl_2_ for studying the influences of metallocene type, solution MAO concentration, polymerization temperature and agitation speed on PP yield, molecular weight, and particle morphology. RHA-supported catalyst exhibited higher polymerization activity than commercial micro-sized silica supported catalyst, and fabricated PP particles with uniform size and shape, which can improve the downstream polymer processability. Me_2_Si(Ind)_2_ZrCl_2_ produced polymer molecules with much greater molecular weight (and longer chain length) but with much smaller number (only one quarter, at the optimum polymerization temperature of 55 °C) than C_2_H_4_(Ind)_2_ZrCl_2_ did, which resulted in the dramatic difference in the shape and size of polymer assembly. At 55 °C, the former had a dumbbell-like shape, but the latter had a spherical shape. The dumbbell-like assembly had a smooth middle section with a width essentially identical to the Batchelor concentration microscale proposed in turbulent mixing theory. The increase of reaction temperature decreased polymer molecular weight and stereoregularity, which resulted in the change of the shape or the decrease of the size of polymer assembly.

## Figures and Tables

**Table 1:** Effects of polymerization conditions on PP properties using RHA-supported Me_2_Si(Ind)_2_ZrCl_2_ catalyst.

Temp. (°C)	MAO (wt%)	Yield (g)	M_n_ (g/mol)	M_w_ (g/mol)	PDI = M_w_/M_n_	*mmmm* (%)
55	0.1	4.0	14,315	49,141	3.4	90
55	0.2	4.7	22,523	51,436	2.3	90
55	0.4	10.7	31,893	81,239	2.5	90
55	0.6	12.1	39,818	109,027	2.7	90
40	0.4	7.3	41,980	113,346	2.7	93
50	0.4	9.1	36,504	84,957	2.3	92
60	0.4	9.5	28,726	65,519	2.3	88
70	0.4	8.9	25,272	53,008	2.1	85

**Table 2:** Effects of supports on propylene polymerization with Me_2_Si(Ind)_2_ZrCl_2_ catalyst.

Support	Polymerization Temperature (°C)	Activity (kg PP/mol Zr.h)	$\bar{\mathrm{Mw}}$ (kg/kg mol)
(1) RHA (this work)	55	4142	81,239
(2) Commercial micro-sized silica [17]	55	440	72,470
